# Transcriptome profiling of lentil (Lens culinaris) through the first 24 hours of Ascochyta lentis infection reveals key defence response genes

**DOI:** 10.1186/s12864-018-4488-1

**Published:** 2018-01-31

**Authors:** Mahsa Khorramdelazad, Ido Bar, Paul Whatmore, Gabrielle Smetham, Vijay Bhaaskaria, Yuedong Yang, Shahla Hosseini Bai, Nitin Mantri, Yaoqi Zhou, Rebecca Ford

**Affiliations:** 1 Environmental Futures Research Institute, School of Natural Sciences, Griffith University, 170 Kessels Rd., Nathan, 4111 QLD Australia; 2 Genecology Research Centre, Faculty of Science, Health, Education and Engineering, University of the Sunshine Coast, Maroochydore DC, 4558 Queensland Australia; 3 Fish Nutrition and Feed Safety, the National Institute of Nutrition and Seafood Research (NIFES), Strandgaten 229, Bergen, 5002 Norway; 4 Faculty of Veterinary and Agricultural Sciences, The University of Melbourne, 142 University St., Parkville, 3053 VIC Australia; 5 Pangenomics Group, School of Sciences, RMIT University, Bundoora, 3083 VIC Australia; 6 Glycomics institute, School of Sciences, Griffith University, 58 Parklands Dr., Southport, Gold Coast, 4215 QLD Australia

**Keywords:** Lentil, *Lens culinaris*, *Ascochyta lentis*, RNA sequencing and transcriptome analysis, *De novo* assembly, Fabaceae, Defence response

## Abstract

**Background:**

Ascochyta blight, caused by the fungus *Ascochyta lentis*, is one of the most destructive lentil diseases worldwide, resulting in over $16 million AUD annual loss in Australia alone. The use of resistant cultivars is currently considered the most effective and environmentally sustainable strategy to control this disease. However, little is known about the genes and molecular mechanisms underlying lentil resistance against *A. lentis*.

**Results:**

To uncover the genetic basis of lentil resistance to *A. lentis*, differentially expressed genes were profiled in lentil plants during the early stages of *A. lentis* infection. The resistant ‘ILL7537’ and susceptible ‘ILL6002’ lentil genotypes were examined at 2, 6, and 24 h post inoculation utilising high throughput RNA-Sequencing. Genotype and time-dependent differential expression analysis identified genes which play key roles in several functions of the defence response: fungal elicitors recognition and early signalling; structural response; biochemical response; transcription regulators; hypersensitive reaction and cell death; and systemic acquired resistance. Overall, the resistant genotype displayed an earlier and faster detection and signalling response to the *A. lentis* infection and demonstrated higher expression levels of structural defence-related genes.

**Conclusions:**

This study presents a first-time defence-related transcriptome of lentil to *A. lentis*, including a comprehensive characterisation of the molecular mechanism through which defence against *A. lentis* is induced in the resistant lentil genotype.

**Electronic supplementary material:**

The online version of this article (10.1186/s12864-018-4488-1) contains supplementary material, which is available to authorized users.

## Background

Lentil (*Lens culinaris* ssp. *culinaris*) is a rich source of protein, minerals and vitamins; and thus plays a staple food role in the diets of vegetarian, vegan and low meat consuming communities. Due to exponential population growth in regions where lentil is a main staple, annual global production has drastically risen, from 0.85 to 5.03 Mt during the last five decades [1]. However, global production and quality is substantially impacted by Ascochyta blight, caused by the necrotrophic fungus *Ascochyta lentis* [2]. Together with the cost of management through fungicides, this pathogen is responsible for an annual estimated loss of $16.2 million AUD in Australia alone [3, 4].

Much research has been conducted to understand *A. lentis* epidemiology, diagnostics, lifecycle, survival and chemical susceptibility [2, 5–8]. This information, together with the adoption of high yielding resistant cultivars, provides the most environmentally friendly and economic strategy for disease management [3]. Relatively few genotypes, containing simply inherited ‘resistance’ to *A. lentis*, have been employed widely in resistance breeding programs on a global scale [8–10]. The lentil industry in Australia is reliant on *A. lentis* resistance from three main sources; two Canadian cultivars *cv.* Northfield and *cv.* Indianhead and the landrace ILL7537, all underpinned by one or two major resistance genes. Recently, the widely adopted resistance derived from *cv.* Northfield (ILL5588), under the control of one [10] or two dominant genes [11], seems to have been eroded through increased pathogen aggressiveness [12]. It is likely that other major resistances may also be under threat through selective adaptation of the pathogen population, and therefore there is an urgent need to understand the key functional genes employed by resistant genotypes to strategically improve the longevity of the defence mechanisms available. The initial step towards this is to identify and characterise the genes involved, however, there is currently limited information on the lentil genome or its interaction with *A. lentis*.

Some initial efforts have been made to explore the genomic and molecular aspects involved in defence to *A. lentis*. Comparative gene expression analysis with a boutique microarray, comprising a limited number of defence-related cDNA probes sourced from other leguminosae species, revealed several genes important in the early resistance reaction of the resistant lentil accession ILL7537 to *A. lentis* at 2, 6, and 24 h post inoculation (hpi) [13]. The suit of differentially expressed (DE) genes uncovered confirmed the biological significance of the early stages in the *A. lentis*–lentil interaction; representative of pathogen recognition (2 hpi), induced defence responses (6 hpi), and necrotic structural defence reactions (24 hpi) [7, 8]. In particular, serine/threonine protein kinases were reported to be a key component of the signalling mechanism required to activate downstream *A. lentis* defence responses, which included an hypersensitive reaction [13]. Histopathology research of *A. lentis* infection and disease progress observed major changes in the lentil physiology at 2, 6, and 24 hpi and depicted those as important checkpoints in the defence response of lentil to *A. lentis* [8]. Lentil plants detect *A. lentis* attack as soon as they come in contact at the host surface or in minutes of invasion. This mainly occurs between 2-6 hpi (first/early phase of oxidative burst) as previously reported [8]. These rapid events are transcription-independent, cause morphological and physiological changes in the infected cells and their surroundings and further transcriptional and post-translational activation of transcription factors takes place. Secondly, a sustained oxidative burst phase that occurs hours after pathogen attack usually associated with the establishment of the defences and the hypersensitive response is carried out. In lentil plants this occurred between 20 and 24 hpi, which may act as a signal for gene activation resulting in secretion of fungal penetration-inhibitory substances into the surrounding plant cell wall to arrest further penetration and spread [8].

Although a good foundation, these results were greatly limited by their dependence on homologous sequences previously discovered as important in other species and pathosystems. Therefore, there remains a large knowledge gap regarding which genes/functional alleles are involved in the early defence pathways of recognition, and biochemical and physiological defence responses in the lentil–*A. lentis* interaction. To bridge this gap, an in-depth molecular study of the interaction is required.

Next generation sequencing and more specifically RNA-Sequencing (RNA-Seq) has become a popular and comprehensively informative approach to monitor wide transcriptional changes during host-pathogen interactions [14–17]. Recently, an RNA-Seq approach was employed to characterise the functional defence response genes of faba bean to *A. fabae* and these included phytoalexins (Dihydrofla-vonol-4-reductase), a chitin elicitor-binding protein (CEBiP), jasmonate O-methyltransferase and an F-box/leucine-rich repeat (LRR) protein, as well as several pathogenesis-related (PR) proteins [17]. Likewise, RNA-Seq revealed that protein kinases such as receptor-like kinases, PR protein classes (2-9, except PR7), diterpene phytoalexin biosynthesis genes, and WRKY transcription factors were involved in the defence of rice to *Ustilaginoidea virens* [15].

A few RNA-seq studies were conducted recently to assemble the expressed transcriptomes of lentil, which provided a good reference for the genes expected to be expressed throughout various tissues and genotypes. However, these focused on different developmental stages and toxic tolerance [18] and marker development [19, 20] and none of them covered the transcriptional changes that occur during a pathogen attack and its counteract defence response. A targeted RNA-Seq approach during lentil–*A. lentis* interactions would be beneficial in better understanding the molecular defence responses of lentil to Ascochyta blight.

Thus, the aims of this study are to use RNA-Seq to identify the genes and gene functions, and predict the molecular pathways employed by a resistant lentil accession in the early recognition and defence to an aggressive isolate of *A. lentis*.

## Methods

### Bioassay and RNA extraction

**Fig. 1:**
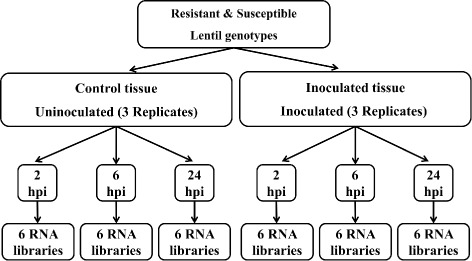
Experimental design of the *Ascochyta lentis* resistant (ILL7537) and susceptible (ILL6002) lentil genotypes used for RNA extraction

Seedlings of lentil genotypes ILL7537 (resistant to *A. lentis* [8]) and ILL6002 (susceptible to *A. lentis* [8]) were grown in a controlled-environment growth room at 20 °C ± 2 °C with 12/12 h dark/light lengths. Three replicates (pots) of five seedlings per 7 cm diameter pot were grown in a light commercial pine bark soil for each of the time points assessed. At 14 d after sowing, seedlings were sprayed until run-off with a 1×10^5^ suspension of *A. lentis* ALP2 isolate condiospores [12] or water as a negative control, according to the method described by Davidson et al. [12]. All seedlings were then placed in the dark for 48 h within a plastic box and adequate humidity was maintained to encourage fungal growth and germination. During this period, the seedlings were harvested and pooled from each pot (replicate) at 2, 6, and 24 hpi. This provided triplicate biological representative reactions for each genotype, at each time point and from both fungal and water inoculated treatments (Fig. 1). Another pot of five seedlings of each genotype that had been inoculated with the isolate was left unharvested and allowed to grow in the growth room at 20 °C ± 2 °C with 12/12 h dark/light lengths to confirm visible disease symptoms after 7−10 d.

At each time of collection, the five seedlings of each replicate were combined and instantly frozen in liquid N_2_. Total RNA was extracted from the whole seedling bulks using the RNeasy plant mini kit along with DNase treatment, according to the manufacturer’s instruction (QIAGEN, Germany). RNA quality and quantity were determined with an Experion RNA analysis kit (Bio-Rad, USA) (Table 1). Subsequently, 6 *μ*g total RNA of each sample were diluted in 50 *μ*L RNAse-free H_2_O and used for cDNA library preparation and transcriptome profiling.

**Table 1:** RNA sample details including quality and concentration measurements

Sample ID	HPI^a^	Cultivar	Treatment^b^	Replicate	RQI^c^	RNA conc. (ng/ *μ*L)
1C	2	ILL6002	Mock	1	9.5	504.93
2C	2	ILL6002	Mock	2	9.5	477.45
3C	2	ILL6002	Mock	3	9.4	359.48
4C	2	ILL6002	Treated	1	9.4	390.33
5C	2	ILL6002	Treated	2	9.4	461.47
6C	2	ILL6002	Treated	3	9.1	214.97
7C	2	ILL7537	Mock	1	9.5	316.84
8C	2	ILL7537	Mock	2	9.4	348.37
9C	2	ILL7537	Mock	3	9.6	281.67
10C	2	ILL7537	Treated	1	9.9	325.65
11C	2	ILL7537	Treated	2	9.4	317.97
12C	2	ILL7537	Treated	3	9.6	211.23
13C	6	ILL6002	Mock	1	10.0	263.26
14C	6	ILL6002	Mock	2	10.0	359.14
15C	6	ILL6002	Mock	3	10.0	157.02
16C	6	ILL6002	Treated	1	10.0	165.98
17C	6	ILL6002	Treated	2	10.0	236.68
18C	6	ILL6002	Treated	3	10.0	260.98
19C	6	ILL7537	Mock	1	10.0	210.47
20C	6	ILL7537	Mock	2	10.0	222.57
21C	6	ILL7537	Mock	3	10.0	296.11
22C	6	ILL7537	Treated	1	10.0	275.24
23C	6	ILL7537	Treated	2	10.0	221.47
24C	6	ILL7537	Treated	3	10.0	275.41
25C	24	ILL6002	Mock	1	9.7	511.10
26C	24	ILL6002	Mock	2	9.7	303.83
27C	24	ILL6002	Mock	3	9.6	467.35
28C	24	ILL6002	Treated	1	9.6	407.76
29C	24	ILL6002	Treated	2	9.5	336.80
30C	24	ILL6002	Treated	3	9.7	263.37
31C	24	ILL7537	Mock	1	9.8	338.72
32C	24	ILL7537	Mock	2	9.7	340.26
33C	24	ILL7537	Mock	3	9.7	550.03
34C	24	ILL7537	Treated	1	9.8	317.72
35C	24	ILL7537	Treated	2	9.8	414.79
36C	24	ILL7537	Treated	3	9.8	481.03

^a^Hours post inoculation

^b^Sample treatment: Treated – inoculation with highly pathogenic isolate of *A. lentis* ALP2; Mock – water spray as a negative control

^c^RNA quality indicator (RQI), as determined by an Experion RNA Analysis System (Bio-Rad)

### Transcriptome profiling

#### Library preparation and RNA-Sequencing

Library preparation and sequencing were performed at the Pangenomics Laboratory, RMIT University, Bundoora, following the methods described in the Ion Proton user’s guide (Thermofisher Scientific, USA). Briefly, mRNA was isolated from the total RNA using Dynabeads mRNA Purification Kit (Thermofisher Scientific, USA). This was followed by enzymatic fragmentation of mRNA to create short reads, <300 base pairs (bp), suitable for the Ion Proton sequencer. The cDNA was synthesised using reverse transcription and a unique barcode was attached to the fragments of each library. The RNA-Seq libraries were prepared using Ion Total RNA-Seq Kit v2 (Thermofisher Scientific, USA) according to the manufacturer’s instructions. Finally, four RNA-Seq libraries were multiplexed and loaded on an Ion Proton^TM^ chip for sequencing. The resulting raw RNA-Seq reads were deposited in the National Center for Biotechnology Information (NCBI) Sequence Read Archive (SRA study accession number: SRP075524).

#### Assembly

Short read sequences from the RNA-Seq were downloaded and processed on the Griffith University ‘Gowonda’ High Performance Computing Cluster using Linux command-line operations. Preliminary quality check of the reads was performed using FastQC (v0.11.2), followed by 3’ and 5’ ends quality trimming and adaptor removal by Trimmomatic (v0.32 [21]), with stringency parameters of SLIDINGWINDOW:4:10 MINLEN:36. The clean and trimmed reads were then *de novo* assembled using Trinity (r20140717 [22]), to establish the full lentil defence-response transcriptome. Assessment of the assembled transcripts was performed by calculating and plotting an ExN50 value against a fraction of the most highly expressed transcripts (Ex). This plot enabled identification of the assembly saturation point, at which the maximum length of N50 was obtained, after removal of the transcripts with minor contribution to the total expression, which are often associated with assembly errors [22, 23].

#### Gene and protein annotations

Open reading frames (ORFs) were predicted from the assembled transcripts using TransDecoder (r20140704); an ORF was considered as complete by the presence of a starting methionine amino acid and an ending stop codon. Transcripts and predicted peptides were annotated by sequence alignment similarity search (BLAST 2.3.0+ [24]) to protein databases (NCBI nr, UniProt, Swiss-Prot and KOBAS, *e*-value <1e-5), and by hidden Markov models protein domain identification (HMMER3.1b [25]) against the HMMER/Pfam protein database (v28.0 [26]). Annotated ORFs were classified into taxonomy groups by extracting the species from their top scoring Blast result. Higher taxonomy levels (family, phyla) were inferred from the NCBI Taxonomy database using the taxize R package [27, 28]. Based on these annotations, Gene Ontology (GO [29]) and Kyoto Encyclopedia of Genes and Genomes (KEGG [30, 31]) terms were assigned to each putative protein. Since *L. culinaris* is not included in the KEGG database of species-specific terms, KEGG orthology terms were used to match a function to each ORF. Furthermore, predictions of putative secretory signal peptides (SignalP v4.1 [32]) and trans-membrane topology (TMHMM v2.0 [33]) were performed. The resulting annotation output files were further processed and cleaned to remove duplicates, select best matching annotation and identify errors or missing values.

To determine how inclusive the assembly was of full length genes, coverage levels of the reconstructed protein-coding ORFs were calculated from the UniRef90 Blast results, as described in the Trinity documentation (https://github.com/trinityrnaseq/trinityrnaseq/wiki/Counting-Full-Length-Trinity-Transcripts). A similar assessment of the representation of single-copy conserved plant orthologues was performed using BUSCO [34].

#### Differential gene expression

The number of reads that mapped to each ORF was estimated by the super-efficient and alignment-free software, kallisto [35]. Preliminary exploratory data analysis of the estimated counts of each sample was performed by variance-stabilising transformation of the raw counts, followed by computing and plotting a between-sample distance matrix and principle coordinate analysis to identify sample-related biases.

The estimated counts were normalised using the Trimmed Mean of M-values (TMM), a normalisation method implemented in the edgeR Bioconductor package (http://bioconductor.org/packages/release/bioc/html/edgeR.html), to account for differences in library size between samples [36, 37]. To represent count data variability, standard error values were calculated per gene, based on triplicate TMM counts (*n* =3) at each genotype/time point, with the exception of the reference genes, where SE were calculated based on all samples and across all experimental groups (excluding the outlier sample 4C, *n* =35). TMM counts of selected defence-related transcript are provided in Additional file 1.

The estimated counts were further analysed by edgeR to identify statistically significant DE ORFs between the experimental groups [38, 39]. Raw *p*-values were adjusted for multiple comparisons by the Benjamini-Hochberg procedure, which controls the false discovery rate [40]. Transcripts were considered to be DE with a |log_2_FC|>1.5 (positive or negative for either over- or under-expression respectively), and an adjusted *p*-value <0.05.

Gene set enrichment analysis of the DE genes was then performed for the GO and KEGG annotations to determine over-represented functional pathways (with a *p*-value <0.01 and *p*-value <0.001, respectively) at each comparison level (for specific genotype, sampling time and treatment combination). Each pathway was further categorised into one of GO’s functional groups (biological processes, cell cycle or molecular function) or KEGG’s functional groups (metabolism, environmental information processing, organismal systems, cellular processes or genetic information processing). The analysis was performed using a custom-written R script (https://github.com/IdoBar/Trinotate_GSEA_plotteR), utilising the goseq R package [41].

#### Quantitative reverse transcription PCR

Real time quantitative reverse transcription PCR (RT-qPCR) was used to validate the expression patterns of selected genes. Three biological replicates from inoculated resistant and susceptible plants at 2, 6, and 24 hpi were collected, instantly frozen in liquid N_2_ and stored at −80 °C until RNA extraction. Samples were pulverised while frozen in liquid N_2_ using a mortar and pestle and total RNA was extracted using NucleoSpin ^*Ⓡ*^ RNA Plant kit along with DNase treatment, according to the manufacturer’s instruction (Macherey Nagel, Germany). Quality and quantity of the total RNA for each sample were determined using gel electrophoresis, NanoDrop^TM^ (Thermo Fisher Scientific, USA) and Qubit (Invitrogen, USA). 0.7 *μ*g of total RNA from each sample was used to synthesise cDNA using PrimeScript^TM^ RT reagent Kit (TaKaRa Bio, Japan), incorporating an additional gDNA removal step. The RT-qPCR was performed in the CFX96 Touch^TM^ Real-Time PCR Detection System (Bio-Rad, USA).

Five antifungal compounds and transcriptional regulators involved in defence were chosen as target genes for the assay: *RBP*, *PR2*, *PR10*, *PGIP*, *ARP* and *DELLA*; and *PP2A* as a reference gene. Primers were designed from the transcriptome sequences for each gene and were tested to ensure acceptable amplification efficiency, specificity, consistency and detection range, based on serial dilution standard curves and melt curves. Amplification efficiencies for each gene were calculated from the coefficients of linear regression equations fitted to the Window-of-Linearity phase of each reaction of the main RT-qPCR assay. The calculated efficiencies were then averaged across all reactions of each gene, as implemented in LinRegPCR (v2017.1) [42, 43]. Details of the primers used for the RT-qPCR assay are listed in Table 2. Each RT-qPCR reaction contained 2 *μ*l of cDNA template (diluted 1:25 from the synthesis reaction), 10 *μ*l SYBR ^*Ⓡ*^ Premix Ex Taq^TM^ (TaKaRa Bio, Japan) and a final primer concentration of 1.6 *μ*M in a final volume of 20 *μ*l. The reactions were performed using the following cycle conditions: an initial 95 °C for 2 min, followed by 38 cycles of 95 °C for 10 s, 50–57 °C for 30 s (depending on the empirically determined optimal melting temperature for each primer pair), 72 °C for 15 s, and a final 5 min extension at 72 °C. All reactions were performed in three technical replicates for each biological sample (*n* =3) at each time point. Inter-run calibrators reactions were included in each plate using a pooled cDNA as template for each of the three reference genes.

**Table 2:** Genes and primers used in qRT-PCR assay

Target gene	Gene function	Primer sequences (5’-3’)	Amplification efficiency^a^	Tm (°C)
*DELLA*	SAR signalling	F: GTCTTCTAATTCAAACCA	1.926 ± 0.027	53
		R: ATATCTGTTTACCCAAGTAA		
*RBP-hnRNP*	Transcriptional factor	F: GAGAAAGATATTTGTTGGAG	1.947 ± 0.024	51
		R: TGATCGTACATTACTACAACA		
*PGIP*	Anti fungal compound	F: TGAAGGTGATGCTTCTATGCT	2.013 ± 0.026	53
		R: GACTCACATCCAACGTTGCT		
*PR2*	Anti fungal compound	F: GGCATGCTGGGAAACAATCT	2.018 ± 0.023	52
		R: TGGCACACCTAACATGAGCT		
*PR10*	Anti fungal compound	F: TGGCACTTCTGCTGTTAGATGGAC	2.017 ± 0.083	50
		R: GGTAATCCATCCAGCCATTTGGAG		
*PP2A*	Reference gene	F: GCCTCATTTGCAGCTGGTTT	2.002 ± 0.025	53
		R: TACTCTCGTTCTAGGGTCCT		

^a^Mean and standard deviation of the amplification efficiency for each gene were calculated from the coefficients of linear regression models fitted to each reaction (value of 2 equivalents to 100% efficiency, see [43])

Amplification data (Cq values) were normalised between plates using Factor-qPCR (v2016.0) [44] which estimates between-plates correction factors based on the inter-run calibrators. DE ratio of each gene between the resistant and susceptible genotypes at each time point was then calculated with the Relative Expression Software Tool (RESTⒸ 2009 [45]), considering the amplification efficiency of each gene [46, 47]. Statistical significance of the DE genes was tested by a randomisation test incorporated in RESTⒸ 2009 and were considered as significant with a *p*-value <0.05.

#### Data analysis

**Fig. 2:**
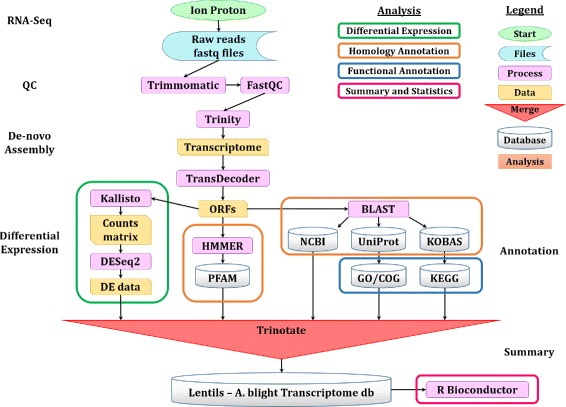
Bioinformatics flowchart of tools and methods used to process and analyse the RNA-Sequencing data and produce the transcriptome

The annotation and expression data files were then combined and loaded onto a lightweight, standalone relational SQLite database (http://www.sqlite.org/), using the scripts provided in the Trinotate pipeline (v3.0.1; https://trinotate.github.io/). This allowed for a fast and easy retrieval of sequences, annotation and expression data using any combination of conditional filtering and ordering. A complete bioinformatics data processing and analysis workflow is presented in Fig. 2.

Statistical analysis and additional data summarising were performed using the R statistical programming language (v3.2.5 [48]). Specifically, relevant data was retrieved from the SQLite database and pre-processed using the dplyr package, and then analysed using various tools from the Bioconductor [49] and the Comprehensive R Archive Network (CRAN, https://cran.r-project.org/) R repositories.

## Results

### RNA-Sequencing and assembly

**Fig. 3:**
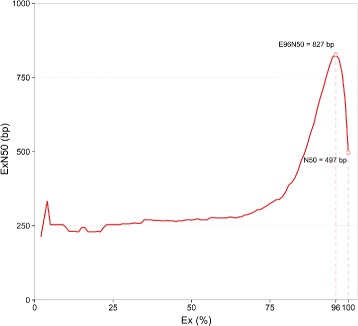
Expression-dependant N50 (ExN50), as calculated against a fraction of the total expressed data (Ex). ExN50 at the point of assembly saturation (96%) and traditional N50 are highlighted

All RNA extracts were of high quality, with an average RNA quality indicator of 9.72, as determined on the Experion (Table 1). A total number of 7.25×10^8^ reads with an average length of 90 bp were produced by the Ion Proton RNA sequencing platform. Of the total number of bases sequenced across all reads (7.90×10^10^ bp), 13% ± 3% in average were trimmed to remove adapters and low quality base calls in each read file. In addition, reads which were too short after adapter trimming and/or had an average low quality bases were dropped, resulting in a total of 6.47×10^8^ clean reads, comprised of 6.86×10^10^ bp high quality bases. Detailed trimming statistics of each read file are provided in Additional file 2. The defence-related transcriptome of lentil was then *de novo* assembled to 317,412 transcripts (total length of 1.45×10^8^ bp), which were grouped into 256,326 trinity ‘genes’, with an N50 = 497 bp. Plotting the ExN50 value against varying levels of cumulative transcript expression (Ex) identified a saturation point of the assembly at 96% of the total expression, giving an improved E96N50 of 827 bp and reducing the effective contig count to 44,007 (Fig. 3). Detailed statistics of the transcriptome assembly are provided in Table 3.

**Table 3:** Lentil–*A. lentis* transcriptome assembly details

Feature	Value
Assembled contigs (n)	317,412
Longest contig (bp)	11,358
Mean contig (bp)	457
Contig N50 (bp)	497
Contig N90 (bp)	236
Contig E96N50 (bp)	827
E96 contigs (n)	44,007
Total contig length (bp)	1.45×10^8^
Assembly GC content (%)	41.74
Total reads (n)	7.247×10^8^
Avg. sequence retention rate^a^ (%)	89.63

^a^Raw sequenced reads which passed quality control measures as determined by FastQC

### Annotation

Multiple ORFs were predicted from each transcript, to a total number of 106,754. Close to 30% of the predicted ORFs presented a minimum of 100 amino acids, a starting codon for Methionine and an ending stop codon and were therefore predicted as ‘complete’. Another 31% of the ORFs were predicted to contain a partial 5’, more than double of those with partial 3’, most likely due to the poly(A) enrichment stage in the library preparation process, which is reported to introduce a bias towards the 3’ end of the transcripts [50].

**Fig. 4:**
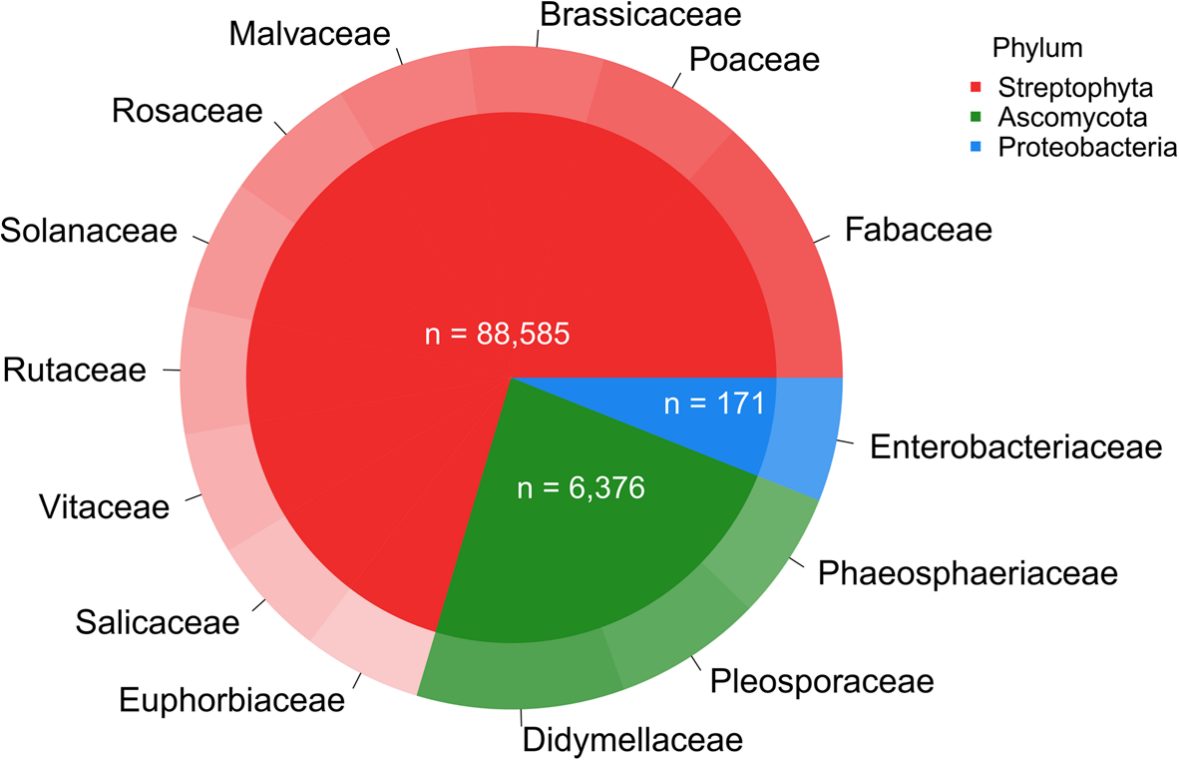
Taxonomy distribution of significant* Blast matches. Annotation was considered as significant with a BitScore >100, pie slices are calculated in logarithmic scale to assist in visualisation

Both the assembled transcripts and predicted ORFs were annotated to known genes, using NCBI’s comprehensive nucleotide (nt) and protein (nr) databases respectively, resulting in significant matches for 70% of the transcripts and over 62% of the ORFs. Taxonomic analysis of the Blast matches revealed that 91.1% of the ORFs matched previously annotated plant genes, 97.6% of those from the legume family (Fabaceae); another 6.5% matched fungi proteins (Ascomycota, mainly from the Didymellaceae family); and another small fraction (1.8%) matched bacterial genes (Fig. 4).

Close to 25% of the Blast annotated ORFs were predicted to cover more than 80% of their target gene’s full length sequence, thus providing strong evidence that the transcriptome assembly contained a reasonable coverage of the genes in the tissues. A similar conclusion arose from the BUSCO analysis, identifying 379 complete single-copy, 339 duplicated and 176 fragmented conserved plant orthologues in the assembly, out of 956 orthologue groups in total (overall coverage of 93.5%).

Additional annotation of protein domains was performed against the Pfam database, assigning significant domains to 56% of the ORFs. Functional annotation of the putative ORFs using GO and KEGG terms, found a significant match to 37 and 33% of the ORFs, respectively, thus adding another layer of annotation at the molecular pathway level. Transmembrane structure and signal peptide predictions were performed as well and added to the complete ORFs annotation database (full details in Table 4).

**Table 4:** Transcript and open reading frame annotation

Annotation	Occurrences (#)	Rate (%)
Transcripts:^a^	317,412	100
NCBI nt^a^	223,246	70.3
UniRef90^a^	138,131	43.5
ORFs:^b^	106,754	100
NCBI nr^a^	93,077	62.4
SwissProt^a^	52,452	49.1
Pfam^c^	60,404	56.6
KEGG^a^	39,919	37.4
GO^a^	34,937	32.7

^a^Blast based annotation was considered as significant with a BitScore >100

^b^ORFs predicted by TransDecoder

^c^HMM-predicted Pfam protein domains were considered as significant with FullSeqScore and FullDomainScore >20

### Differential gene expression

#### Exploratory data analysis

**Fig. 5:**
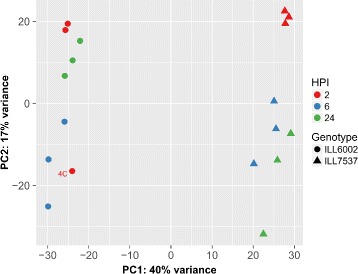
Principal component analysis of the variance-stabilized estimated raw counts. Samples are categorized by Genotype (as marker shapes) and Hours post inoculation (HPI, marker colour)

An exploratory data analysis of the estimated counts of reads that mapped to each ORF clearly showed that the main difference between the samples were derived from the genotype variable, which explained 40% of the total detected variation. Sampling time (2, 6, and 24 hpi) contributed another 17% of the variability (Fig. 5).

The three replicates of each sampling group clustered well together, with the exception of sample 4C of the susceptible genotype (ILL6002, round markers in Fig. 5), which clustered with the 6 hpi samples of the same genotype. This sample was identified as an outlier and was excluded from downstream DE analysis.

#### Gene set enrichment analysis

**Fig. 6:**
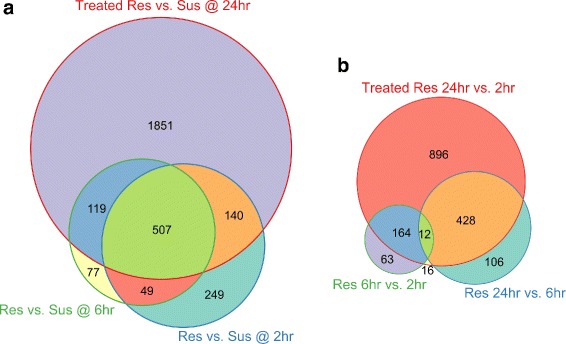
The number of unique and common differentially expressed genes between the subgroups of inoculated samples (time post inoculation and genotype). Comparison of inoculated resistant *vs.* susceptible genotypes at each time point (2 hpi, 6 hpi and 24 hpi, **a**); and within the inoculated resistant (ILL7537) genotype samples between the different time points (**b**). Circle area is plotted to scale (Euler diagram) when geometrically possible

**Fig. 7:**
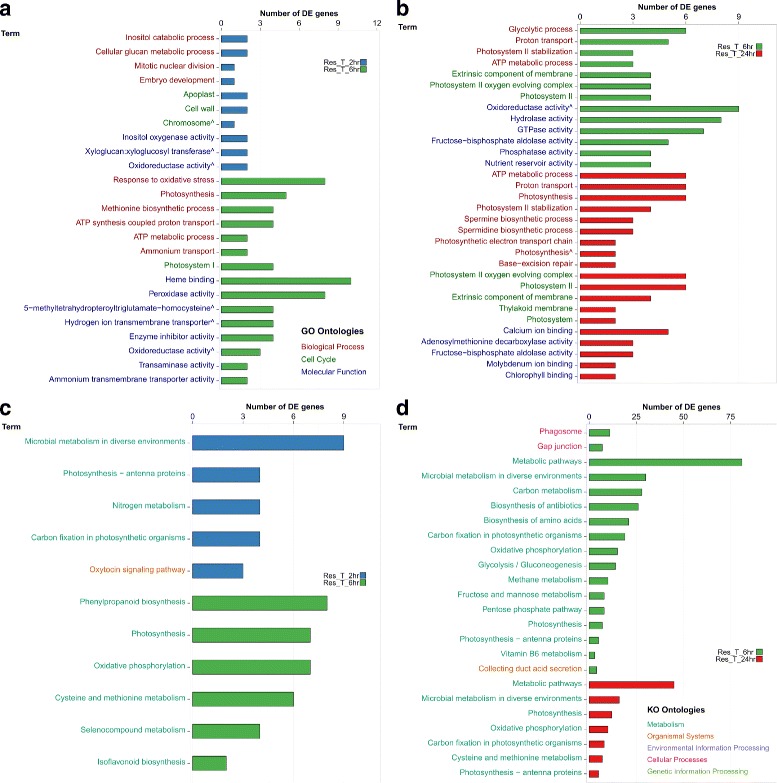
GO and KEGG pathway enrichment analysis, based on over-expressed DE genes at each time point (2, 6, and 24 hpi) in the resistant lentil genotype ILL7537. GO pathway enrichment at 2 and 6 hpi (**a**) and 6 and 24 hpi (**b**); KEGG pathway enrichment at 2 and 6 hpi (**c**) and 6 and 24 hpi (**d**)

The number of putative genes (based on ORF prediction) that were DE, with |*log*_2_*FC*|>1.5 and FDR <0.05, was determined within each comparison, demonstrating once more that the most noticeable DE was found between the resistant genotype and the susceptible one, in particular at 24 hpi, with 2617 DE genes (Fig. 6). A substantial number of genes (507) were commonly DE between the two genotypes, regardless of sampling time. The DE genes were then grouped into functional pathways by their assigned GO and KEGG terms. Comparison of GO enrichment analysis of DE genes in the resistant genotype between 2, 6, and 24 hpi identified high representation of over-expressed genes involved in pathogen recognition, signalling at 2 hpi compared with 6 hpi. In contrast, high proportions of genes associated with anti-fungal compounds, plant cell wall organisation and construction, as well as transcriptional regulators, were observed at 6 hpi (Fig. 7a). At 24 hpi, the majority of DE gene were associated with regulatory functions in plant stress tolerance, antimicrobial compounds and photosynthesis pathways (Fig. 7b).

KO pathway enrichment analysis of the same time points provided a similar picture to the GO enrichment analysis (Fig. 7c-d). High representation of genes involved in microbial, carbon and nitrogen metabolism and signalling pathways were identified at 2 hpi; a substantial number of genes involved in metabolic pathways, photosynthesis, and to a lesser extent, defence response genes at 6 hpi and an increased enrichment of photosynthesis related genes at 24 hpi.

#### Primary defence response (2 hpi)

**Fig. 8:**
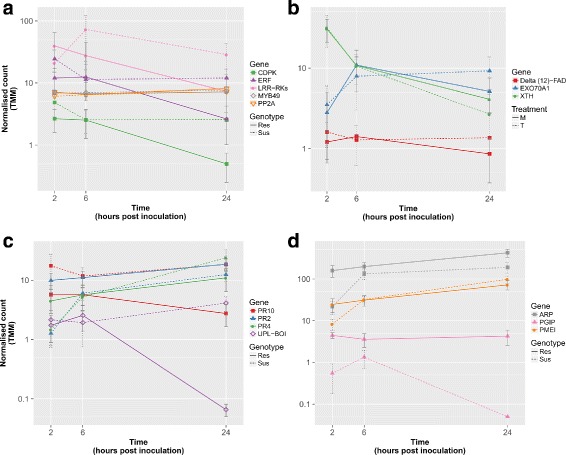
Expression levels of selected genes with exceptional DE trends in the earlier stages of the defence response to *A. lentis* in ILL7537 and ILL6002 over 2, 6, and 24 hpi. Expression levels of the following genes are presented: *CDPK*, *ERF* and *LRR-RK*, with *PP2A* and *MYB49* as examples of stable reference genes (**a**); *Delta (12)-FAD*, *EXO70A1* and *XTH* (**b**); PR genes and *UPL-BOI* (**c**); *ARP*, *PGIP* and *PMEI* (**d**). A full line represents the expression level in the resistant genotype ILL7537 and the dashed line represents the expression level of the gene in the susceptible genotype ILL6002. Y-axis is in logarithmic scale, error bars represent standard error values between replicates

Analysis of DE transcripts among the resistant (ILL7537) and susceptible (ILL6002) genotypes at 2 hpi, compared to those at 6 and 24 hpi identified transcripts with matching annotation to several gene families. Specifically, genes from the protein kinase-like family, known to be involved in pathogen recognition and early stage of signalling [51], were moderately over-expressed at 2 hpi (Fig. 8a). A member of this family is the LRR receptor-like kinase (*LRR-RK*), which demonstrated its highest expression levels in the resistant genotype at 2 hpi, with TMM = 40 and a gradual decrease down to TMM = 7.3 at 24 hpi (log_2_FC=2.45). The expression of *LRR-RK* at 2 hpi in ILL6002 was lower than in ILL7537 (TMM = 20.9), however, it then increased dramatically at 6 hpi (TMM = 73), before decaying back to base levels at 24 hpi, as in ILL7537 (Fig. 8a).

Calmodulin domain protein kinase-like (*CDPK*) was also moderately expressed at 2 hpi and 6 hpi, with a TMM of 2.6, dropping at 24 hpi to just under 0.5 at a log_2_FC of 2.5 in the resistant genotype (Fig. 8a). Slightly higher expression levels were noticed in the susceptible genotype at 2 hpi (TMM = 4.8), dropping to a stable level of 2.5 at 6 and 24 hpi. Interestingly, a very similar expression pattern, with approximately 5x times higher TMM values compared with *CDPK*, was found for Ethylene-responsive transcription factor (*ERF*) across all time points and genotypes (Fig. 8a).

Xyloglucan endotransglucosylase/hydrolase (*XTH*), encoding an enzyme involved in cell wall elongation and restructuring [52], was most highly expressed at 2 hpi in the susceptible genotype, with TMM = 61 (Fig. 8). *XTH* expression levels then rapidly decreased at 6 hpi to 15.3 and then more gradually to 5.4 at 24 hpi (log_2_FC of 2 and 3.5, respectively). A similar expression pattern was observed in the resistant genotype, at approximately half the levels shown in the susceptible genotype at each time point (Fig. 8b).

A unique expression pattern was identified for Exocyst subunit 70A1 (*EXO70A1*), a structural gene involved in papilla formation [53]. This gene was expressed at moderate levels (TMM = 3.47) at 2 hpi in the resistant genotype and then increased at 6 hpi and slightly more at 24 hpi. In contrast, in the susceptible genotype, *EXO70A1* was expressed at low levels at 2 hpi (TM <1), with a steep incline at 6 hpi to TMM = 12.8, superseding the expression levels at the resistant genotype, before declining again at 24 hpi to TMM of 2.5, with a log_2_FC of 4.44 (Fig. 8b). Delta (12)-FAD demonstrated modest expression levels of just over 1 TMM in the resistant genotype, but was still up-regulated comparing its expression in the susceptible lentil genotype, which was negligible (Fig. 8b).

A noticeable expression pattern was observed for pathogenesis related proteins PR-2–O-glycosyl hydrolase family 17 (*PR2*) and PR-4–Thaumatin-like (*PR4*) proteins. *PR2* was over-expressed in ILL7537 at all time points, from which the most significant differential over-expression between the genotypes occurred at 2 hpi with a log_2_FC of 3. *PR4* demonstrated similar expression to *PR2* at 2 hpi with a log_2_FC of 1.65 between the resistant and susceptible genotypes, however, its expression in the susceptible genotype then increased to match the same expression level as the resistant genotype at 6 hpi and even further at 24 hpi (Fig. 8c).

Plant invertase pectin methylesterase inhibitor (*PMEI*) was slightly over-expressed in comparison to the susceptible genotype at 2 hpi, with a log_2_FC of 1.6, followed by a gradual increase in expression at 6 hpi and 24 hpi in both genotypes (Fig. 8d). Polygalacturonase inhibitor (*PGIP*), which encodes a plant extracellular leucine-rich repeat protein [54], was over-expressed in the resistant genotype at 2 hpi with a TMM of 4.3 and log_2_FC of 3.14 in comparison with the susceptible genotype. Another fungal inhibitor with high expression across all experimental groups is the Auxin-repressed protein (*ARP*). *ARP* was found to be DE between the genotypes at 2 hpi, with a normalised count of 158 in the resistant genotype compared with 21.6 in the susceptible, resulting in a log_2_FC of 2.9 (Fig. 8d).

#### Secondary defence responses (6 hpi)

**Fig. 9:**
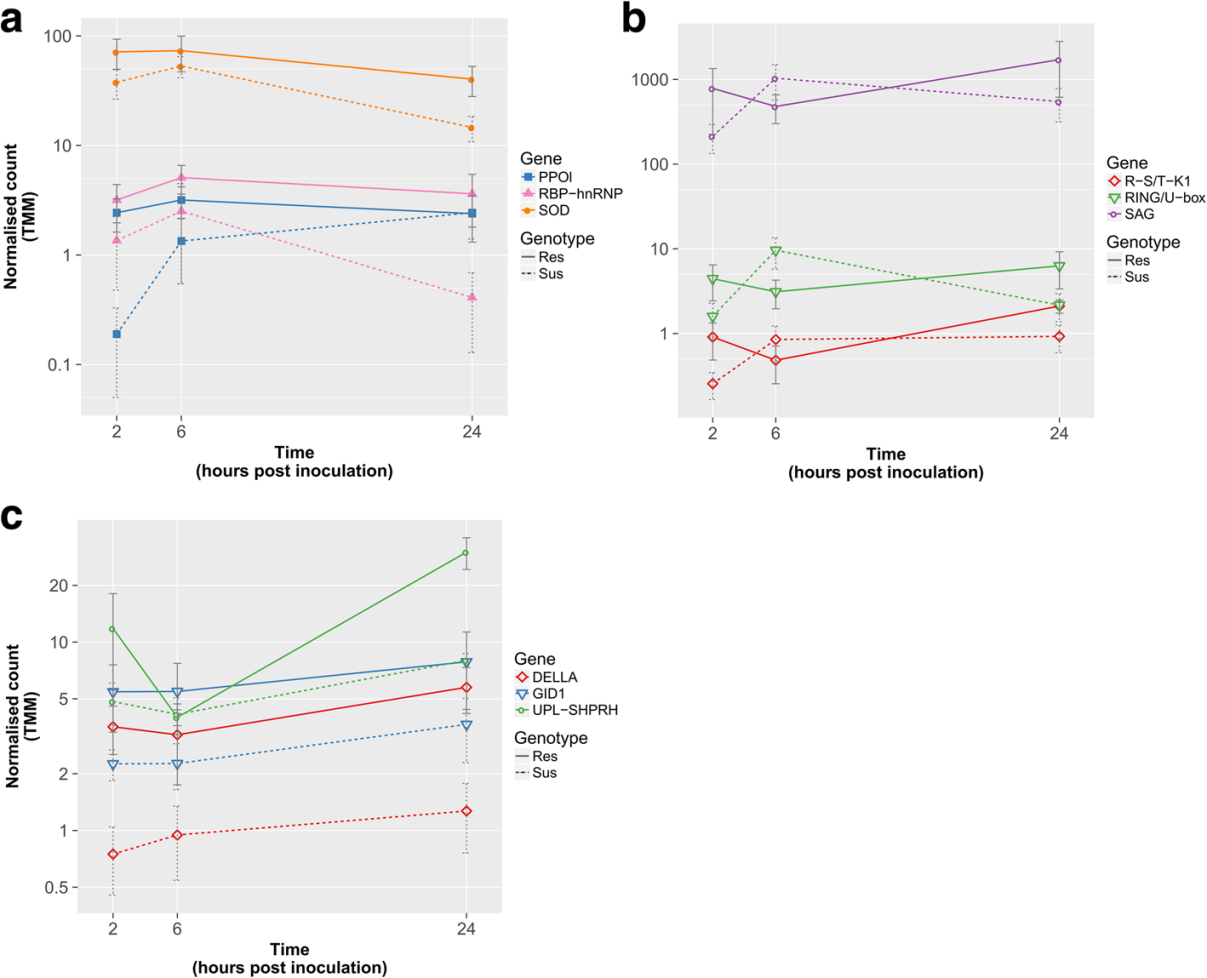
Expression levels of selected genes with exceptional DE trends in the earlier stages of the defence response to *A. lentis* in ILL7537 and ILL6002 over 2, 6, and 24 hpi. Expression levels of the following genes are presented: *R-S/T-K1*, *RING/U-box* and *SAG* (**a**); *R-S/T-K1*, *RING/U-box* and *SAG* (**b**); *DELLA*, *GID1*, *NB-ARC* and *UPL-SHPRH* (**c**). A full line represents the expression level in the resistant genotype ILL7537 and the dashed line represents the expression level of the gene in the susceptible genotype ILL6002. Y-axis is in logarithmic scale, error bars represent standard error values between replicates

Differential gene expression of resistant genotype at 6 hpi compared to 2 and 24 hpi and to susceptible genotype at the same time-point revealed additional functional genes. A significant increase in the expression from 2 to 6 hpi was noticed in the susceptible genotype for the anti-fungal genes mentioned at the primary defence stage (*PMEI*, *PGIP*, *ARP*; Fig. 8d). An expression pattern similar to that of the anti-fungal genes mentioned above was observed for Laccase diphenol oxidase (*PPOl*), showing a slight up-regulation at 6 hpi in the susceptible genotype, with a normalised count of 3 and log_2_FC of 3.2 compared to 2 hpi and overall up-regulation in the resistant genotype, mainly at 2 hpi (Fig. 9a).

Bet v 1 domain PR-10 (*PR10*) protein demonstrated quite an opposite expression pattern in the resistant and the susceptible genotypes. In the susceptible genotype, *PR10* was fairly highly expressed at 2 and 24 hpi with TMM of 17.3 and 18.3, dropping slightly to 11.6 at 6 hpi (Fig. 8c). In contrast, the expression of *PR10* in the resistant genotype was highest at 2 hpi (TMM = 5.6), before dropping to half of that at 24 hpi, with overall lower expression levels than in the susceptible genotype at all time points, with log_2_FC of 2.7, 2.1 and 2.8 at 2, 6, and 24 hpi, respectively. Quite similar expression pattern was observed for Botrytis susceptible interactor E3 ubiquitin protein ligase (*UPL-BOI*), with a peak in its expression at 6 hpi in the resistant genotype with TMM of 2.5, dropping to almost non-existent levels at 24 hpi. In contrast to the resistant genotype, *UPL-BOI* was up-regulated at 24 hpi with a TMM = 4 (Fig. 8c).

Superoxide dismutase (*SOD*) showed high expression levels at 2 and 6 hpi in the resistant genotype with TMM >70, dropping to TMM = 40 at 24 hpi, with matching trend at the susceptible genotype, though with lower expression levels (log_2_FC of about 1.5 at 24 hpi) (Fig. 9a). An RNA binding motif of the heterogeneous nuclear ribonucleoproteins class (*RBP-hnRNP*) also showed its highest expression at 6 hpi in ILL7537, with a TMM of 5. *RBP-hnRNP* showed significant over-expression in the resistant genotype at all time points, but chiefly at 24 hpi with a log_2_FC of 3.3 (Fig. 9a).

#### Tertiary defence responses (24 hpi)

The expression levels of senescence-associated gene (*SAG*) were very high, peaking at 24 hpi in the resistant genotype with a normalized count of 1714 and log_2_FC of 3 compared with the susceptible one at the same time point (Fig. 9b). Interestingly, the expression of *SAG* in the susceptible genotype was a mirroring image of the resistant one, with completely opposite pattern, showing lowest expression levels at 2 hpi (TMM = 213), increasing to a maximum at 6 hpi (TMM >1000) and dropping again at 24 hpi to a TMM of approximately 550. Two other genes, the E3 Ubiquitin ligase *RING/U-box* and Receptor-like Serine/Threonine kinase 1 (*R-S/T-K1*), expressed with exactly the same pattern of the resistant *vs.* susceptible genotypes as *SAG*, however, with much lower expression levels (Fig. 9b).

Gibberellin signalling *DELLA* protein and Gibberellin receptor *GID1* exhibited very similar expression trends: a gradual increase in expression with time and over-expression in the resistant genotype, with an average log_2_FC across all time points of 2.13 and 1.3, respectively (Fig. 9c). *GID1* expression was approximately 2.5x higher than that of *DELLA* at all time points. The expression of *NB-ARC* domain disease resistance protein demonstrated similar trend, gradually increasing throughout the experiment and up-regulated in the resistant genotype across all time points, mainly at 24 hpi with a TMM of 5 compared to 2 in the susceptible genotype and a log_2_FC of 1.85 (Fig. 9b). The E3 Ubiquitin ligase *SHPRH* also demonstrated similar gradual increase in expression in the susceptible genotype, with moderate expression levels at 24 hpi with a TMM of 8, compared to its much elevated expression in the resistant genotype at the same time point, with a TMM of 30, giving a log_2_FC of 1.9.

#### Reference genes

A number of genes showed stable expression patterns at moderate levels across all time points and experimental groups assessed. A Myb-related transcription factor-like protein (*MYB49*) showed the most stable expression, with an average TMM normalised count of 7.00 ± 0.38, followed by a protein phosphatase 2A (*PP2A*) and a *P72 DEAD box*, displaying average TMM of 6.90 ± (44) and 8.3 ± (5), respectively (Fig. 8a). These would potentially be useful for future reference-based comparisons of DE of specific gene target studies, such as RT-qPCR.

### RT-qPCR validation

The DE patterns of five genes involved in the lentil defence to *A. lentis* were validated using RT-qPCR and compared to those obtained from the RNA-Seq transcriptome analysis. *PP2A* demonstrated a constant expression across all time points and both genotypes, with a standard deviation of just ±0.62 in its Cq values, similar to its RNA-Seq derived expression, described in the previous section and therefore it was used as a reference gene in the RT-qPCR analysis.

**Fig. 10:**
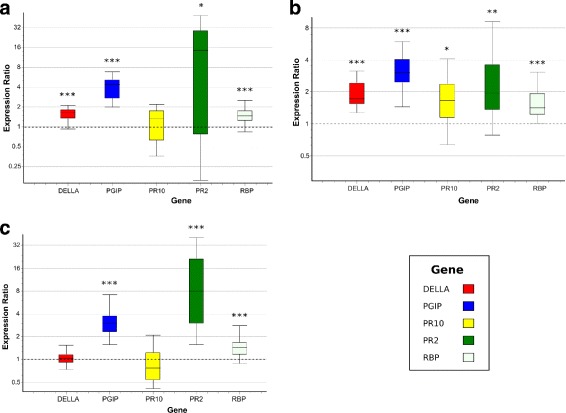
Expression ratios between resistant (ILL7537) and susceptible (ILL6002) lentil genotypes, measured by RT-qPCR. Expression ratios of selected defence-related genes at 2, 6, and 24 hpi (**a**, **b**, **c**, respectively). Asterisks denote statistic significance (DE ≠ 1), with the following *p*-values: * <0.05, ** <0.01, *** <0.005

The target genes, encoding antifungal compounds and transcriptional regulators, consisted of *PR2*, *PR10*, *RBP*, *DELLA* and *PGIP* and the DE of each gene between the resistant (ILL7537) and susceptible (ILL6002) genotypes was measured at each time point. The DE results revealed similar expression patterns to those measured by RNA-Seq across the three time points, with significant over-expression at the ILL7537 genotype, with the exception of *PR10* (under-expressed in ILL7537 according to the RNA-Seq analysis), whose expression was slightly over-expressed at 6 hpi and slightly under-expressed (although not significantly) at 2 and 24 hpi (Fig. 10).

## Discussion

### Exploratory data analysis

The clear separation of the resistant genotype samples from the susceptible genotype samples supports the reported genetic distance between the genotypes [55]. Other than the outlier sample (4C), all samples clustered along with their respective replicates, validating the reproducibility of the assay and the different expression patterns of each experimental group.

### Gene set enrichment analysis

Significant enrichment of metabolic regulation pathways was observed and these included changes to photosynthesis genes. This enrichment is likely to be directly related to the bioassay method, as both treated (inoculated with fungus) and control (sprayed with H_2_O only) plants were placed in darkness after fungal inoculation for 24 h in order to enhance pathogen germination on the plant surface [56, 57]. In addition to the darkness treatment, *A. lentis* causes severe leaf and stem lesions and eventually wilting, which adversely affects the photosynthesis capabilities of the plant and therefore would have contributed to changes in photosynthesis related transcripts [58, 59].

### Gene expression

The DE defence-related genes characterised in this study could be divided into six groups, based on their function and timing of expression relevant to the defence against the *A. lentis* infection.

#### Recognition and early signalling

In the primary resistance response, two protein kinases were identified to play key roles in pathogen recognition and early signalling: a leucine-rich repeat receptor kinase (LRR-RK) and a calmodulin domain protein kinase (CDPK). The leucine-rich repeat motif in LRR-RK and serine/threonine kinase-like domain in CDPK are known to be involved in pathogen invasion recognition and signalling, respectively, to trigger the defence response during host-pathogen interaction [15, 51, 60, 61].

In a related defence-response pathway, activation of ethylene response factor (ERF) positively regulates the expression of Ca^2+^/Calmodulin-dependant protein kinase (*Sl*CCaMK), recently described as a key signalling gene in resistance of tomato to *Sclerotinia sclerotiorum* [62, 63]. Considering that *CDPK* demonstrated an expression pattern highly matching that of *ERF*, along with its sequence and structural similarity to *CCaMK* [64, 65], this suggests the *CDPK-like* transcript detected in the present study as a key early signalling molecule in lentil, following the recognition of *A. lentis* invasion. The *LRR-RK*, which showed similar expression pattern to *ERF* and *CDPK* in the resistant genotype, is likely to trigger the lentil CDPK-like gene for downstream signalling by activating ERF [51, 61, 66]. The reduced levels of *LRR-RK* that were observed in the susceptible genotype (ILL6002) at this early stage, followed by an increase in its expression at later stages, in correlation with elevated levels of *ERF* and *CDPK*, suggests a late and over-stressed defence response in this genotype.

#### Structural defence response

**Fig. 11:**
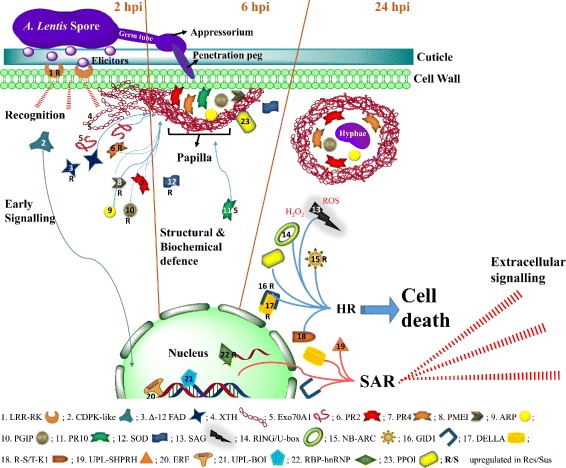
Defence-related molecules involved in response of lentil to *A. lentis* during the first 24 h

Subsequent to early signalling, structural and biochemical responses to the invading *A. lentis* hyphae were detected. As a physical barrier, lentils often accumulate structural compounds at the point of penetration, also known as papilla formation [7, 8] (Fig. 11). Accumulation of xyloglucan endotransglucosylase/hydrolase (XTH) and its function in elongation and restructure of cell walls as part of a physical barrier, was reported in the response of tomato to *Cuscuta reflexa* [67]. Detection of elevated transcript levels of *XTH* at 2 hpi in both genotypes in the current study, suggests early response to the inoculation and preparation for papilla formation. One more structural gene, encoding laccase diphenol oxidase (*PPOl*), was over-expressed in the resistant genotype at 2 hpi and to a lesser extent at 6 hpi, the timing of actual papilla formation [7]. PPOl prevents in vivo pathogen spread [68] by cross-linking cell wall polymers and triggering a hypersensitive response through production of free radicals [69]. Another gene associated with papilla formation in response to spore germination is the gene encoding for Exocyst subunit 70A1 family protein (Exo70A1), through its cellular polarity regulating function [53, 70, 71]. The under-expression of *Exo70A1* in the susceptible genotype at 2 hpi was compensated by a steep incline in expression at 6 hpi. The expression levels of both *Exo70A1* and *PPOl* were low at 2 hpi in the ILL6002 susceptible genotype and then increased to “catch up” with the resistant genotype only at 6 hpi, suggesting a delayed recognition and response to *A. lentis* compared to ILL7537.

Another gene which was over-expressed in the resistant genotype and practically missing from the susceptible one, thus seemingly playing a part in the structural defence response to *A. lentis*, is *Delta (12)-FAD*. FAD proteins are essential for maintaining cellular function and influence a variety of processes such as the regulation of membrane fatty acid profiles in different tissues, different developmental stages, and in response to abiotic and biotic stresses [72]. Accumulation of *Delta (12)-FAD* mRNA was previously demonstrated in parsley cells following treatment by a fungal elicitor, Pep25, and was reported to be involved in the complex defence response by reinforcing existing cell walls [73].

#### Biochemical defence response

During a biochemical defence response to *A. lentis*, lentils use anti-fungal compounds including pathogenesis-related (PR) proteins and reactive oxygen species (ROS), as was previously described [8, 13]. In the present study, members of three families of PR proteins were identified to be significantly DE in ILL7537 in response to *A. lentis* when compared to ILL6002. The three PR protein families include: PR2, 4 and 10 (Fig. 11 and Table 5). PR2 proteins catalyse the hydrolytic cleavage of 1,3- *β*-D-glucosidic linkages in *β*-1,3-glucans present in the fungal cell walls, and cause cell lysis and death in fungi [74]; and were shown to play a vital role in defence against pathogenic fungi such as *Fusarium oxysporum* in chickpea [75]. PR2 protein is involved not only in hydrolysis of fungal-cell components, but it also releases elicitors from the walls of fungi, which in turn may be recognised by plant receptor molecules and stimulate various downstream signalling and defence responses [76]. PR4 protein disrupts fungal cell polarity and inhibits its growth, reacting with nascent chitin at the hyphal tip [13] and its involvement in lentil defence against *A. lentis* was previously characterised in some depth [77].

**Table 5:** Key genes in lentil defence-response to *A. lentis*

Functional groups	Defence gene name	Gene abbreviation	Dominant genotype^a^
Recognition: Receiving & signalling molecules	Calmodulin domain protein kinase-like	*CDPK-like*	
	LRR receptor-like kinase	*LRR-RK*	ILL6002
	Receptor-like serine/ threonine protein kinase 1	*R-S/T-K1*	
	NB-ARC domain disease resistance protein	*NB-ARC*	ILL7537
	Botrytis susceptible interactor E3 ubiquitin protein ligase	*UPL-BOI*	
Early structural response: Papilla formation	Exocyst subunit 70A1 protein	*EXO70A1*	
	Laccase diphenol oxidase	*PPOl*	
	Delta (12)-fatty-acid desaturase	*Delta (12)-FAD*	ILL7537
	Xyloglucan endotransglucosylase/ hydrolase	*XTH*	ILL6002
Early Biochemical response: Pathogenesis-related & anti-fungal compounds	PR protein 2 – O-glycosyl hydrolase	*PR2*	ILL7537
	PR protein 4 – Thaumatin family	*PR4*	
	PR protein 10 – Bet V I type	*PR10*	ILL6002
	Plant invertase pectin methylesterase inhibitor	*PMEI*	
	Auxin-repressed protein	*ARP*	ILL7537
	Polygalacturonase inhibitor	*PGIP*	ILL7537
	Superoxide dismutase	*SOD*	ILL7537
Regulation of defence-related transcripts: DNA & RNA binding proteins	Ethylene response factor	*ERF*	
	Heterogeneous nuclear ribonucleoprotein RNA binding motif	*RBP-hnRNP*	ILL7537
	Senescence associated gene	*SAG*	
Hypersensitive response: Cell death & SAR	RING/U-box E3 ubiquitin protein ligase	*RING/ U-box*	
	Gibberellin signalling DELLA protein	*DELLA*	ILL7537
	Gibberellin receptor	*GID1*	ILL7537
	E3 ubiquitin-protein ligase SHPRH	*UPL-SHPRH*	

^a^Dominant genotype was specified for genes whose expression was generally up-regulated at all time points in one of the genotypes: ILL7537 – Resistant, ILL6002 – Susceptible

In contrast, PR10, which was over-expressed in the susceptible genotype at all time points, is known to exhibit RNase activity on invading intracellular fungal hyphae [78] and was shown to accumulate and correlate with increasing fungal biomass [79]. Therefore, the expression of *PR10* indicates that the susceptible genotype was challenged by higher fungal load throughout the experiment. The Botrytis susceptible1 interactor (*UPL-BOI*) gene showed a similar expression trend to that of *PR10*. UPL-BOI is an E3 ubiquitin protein ligase, which regulates pathogen resistance responses in Arabidopsis to *Botrytis cinerea* [80]. Therefore, UPL-BOI may be involved in expression regulation of *PR10* in lentil in response to *A. lentis*.

Other DE genes with known antifungal activity were plant invertase pectin methylesterase inhibitor (*PMEI*), polygalacturonase inhibitor (*PGIP*), and auxin-repressed protein (*ARP*), all significantly up-regulated at 2 hpi in ILL7537 following *A. lentis* inoculation. PMEI reduces the susceptibility of the plant wall to fungal endopolygalacturonases and was previously reported to aid in Arabidopsis defence to *B. cinerea* and *Pectobacterium carotovorum* [81, 82], in wheat defence to *Bipolaris sorokiniana* and *F. graminearum* [83] and in pepper defence to *Xanthomonascampestris pv. vesicatoria* [84]. Similarly, PGIP limits the destructive potential of fungal polygalacturonases through specific binding and inhibition of them [85]. PGIP also increases the production of oligogalacturonides, leading to the accumulation of phytoalexin, an antibiotic, in plant tissue [86], as reported in tomato defence to *B. cinerea* [87] and *Lathyrus sativus* defence to *Aspergillus niger* and *Rhizopus* spp. [88]. Meanwhile, ARP inhibits pathogens by either producing auxin or manipulating host auxin [89] and was shown to be involved in rice defence to *Magnaporthe grisea* and *Striga hermonthica* [90]. Together, PMEI, PGIP and ARP, thus appear to operate in the lentil ILL7537 resistant genotype to control the spread and growth of *A. lentis* in the early stages following invasion.

#### Hypersensitive reaction and cell death

A hypersensitive reaction in the infected plant is triggered by an oxidative burst and is characterised by an increase in free radicals that leads to localised cell death. As previously determined through histopathological and molecular studies, this defence response is likely to be important in resistant lentil genotypes [7, 8, 13]. This was further demonstrated in the current study through DE of the senescence associated gene (*SAG*), which highly over-expressed at 24 hpi in ILL7537. SAGs are induced by free radicals, such as ROS and H_2_O_2_, leading to a programmed cell death [91, 92] and have been implicated in the defence of Arabidopsis to several biotrophic pathogens [93]. This is the first report of SAG involvement in defence to a necrotrophic pathogen. A similar expression trend was observed for the gene encoding NB-ARC domain disease resistance protein (*NB-ARC*), a domain of NB-LRRs that regulates signal transduction leading from recognition to hypersensitive response-signalling and cell death [94, 95]. The gene encoding RING/U-box protein (*RING/U-box*), which is another E3 ubiquitin ligase, was also significantly over-expressed at the same time points as *SAG* and *NB-ARC*. RING/U-box proteins are involved in the hypersensitive defence response of tomato to *Phytophthora infestans* [96] and in pathogen-instigated programmed cell death of Tobacco [97]. Therefore, it can be assumed that SAG, NB-ARC and RING/U-box are key control genes for the hypersensitive response of lentil to *A. lentis*, triggered in an effort to contain the invading pathogen.

Following the hypersensitive response, the plant activates mechanisms to protect its healthy cells from further damage, as demonstrated by the over-expression of superoxide dismutase (*SOD*) in ILL7537 genotype and its elevated expression levels at 6 hpi. This serves to regulate the redox status of the plant cells and protect from the oxidative burst and generated ROS [98].

#### Systemic acquired resistance (SAR)

Systemic acquired resistance (SAR) is the long distance signalling of pathogen recognition and defence induced by signal molecules and plant hormones. This process may confer long-lasting protection against an invading pathogen and together with the hypersensitive reaction, signal the last stage of early defence responses [99]. In the defence transcriptome of lentil to *A. lentis*, three putative SAR-associated genes, Gibberellin signalling DELLA protein (*DELLA*), Gibberellin receptor (*GID1*) and an E3 ubiquitin ligase (*UPL-SHPRH*), had similar DE patterns with highest transcription levels observed at 24 hpi and general over-expression in ILL7537. DELLA proteins promote defence to necrotrophic fungal pathogens by activating jasmonic acid/ethylene-dependent defence responses [100] and by regulating ROS levels [100, 101]. GID1 binds to DELLA, which then leads to ubiquitination and degradation of DELLA during SAR signalling [102, 103]. Meanwhile, E3 UPLs are involved in recognition, signalling, hypersensitive reaction and cell death mechanisms in Arabidopsis [104, 105] and rice [106–109]. Therefore, considering their expression trends in previous and current studies, we suggest that DELLA, GID1 and UPL-SHPRH proteins are involved in SAR signalling in the early lentil defence mechanisms to *A. lentis*.

#### Transcription regulators

Since the differences observed between the resistant and the susceptible genotypes in the current study were transcriptomic changes, it is also useful to observe changes in the expression of transcription regulators. Therefore, a DE DNA binding transcription factor was identified, encoding Ethylene response factor (ERF). ERF stimulates the expression of PR proteins gene promoters and as discussed earlier, it increases the expression level of early signalling molecules [62, 64, 66]. The decline in *ERF*’s expression in the resistant genotype at 24 hpi, may indicate the success of the early defence responses mentioned previously for ILL7537, whereas the late response of ILL6002 required continuous elevated expression of the defence-related proteins.

Moreover, an RNA binding protein of the heterogeneous nuclear ribonucleoproteins (RBP-hnRNPs) class, up-regulated in ILL7537, is a member of an RNA binding transcriptional factors family that regulate post-transcriptional gene expression [110]. These RNA binding transcriptional factors play key role in regulating transcription of genes in response to biotic and a-biotic stress in plants [111, 112]. The hnRNP-like protein *At*GRP7 have been shown to play a regulatory role on SOD and ARP in Arabidopsis, by affecting the processing of their regulatory microRNAs [113]. *At*GRP7 was further suggested to be involved in defence against fungal and bacterial infections in Arabidopsis by interacting with specific LRR-RKs [114]. The *hnRNP* that was identified in this study was up-regulated in ILL7537, in a pattern matching that of *SOD* and *ARP* (Figs. 8 and 9), suggesting it performs a similar function to *At*GRP7.

### Reference genes and RT-qPCR validation

Protein phosphatase 2A (*PP2A*) and *P72 DEAD box* RNA helicase have previously been used as reference genes for relative DE analysis in plant-pathogen interaction studies [115, 116]. Their stable expression across all the treatments and samples in the current study, along with the RT-qPCR DE results of the selected defence genes, which overall conform to those found in the RNA-Seq analysis, validate the reliability and reproducibility of the RNA-Seq quantification and downstream DE analysis.

*MYB49*, whose exact function is yet to be discovered, is a member of a diverse family of DNA-binding transcription factors [117, 118]. Considering its most stable expression in our data, which supersedes *PP2A* and *P72 DEAD box*, it should also be considered as a reference gene in similar plant-pathogen DE studies.

## Conclusions

The results of the current study are highly concordant with the physiology of the interaction between lentil and *A. lentis* and similar pathosystems [7]. Overall representation of the molecules involved in the defence response of lentil to *A. lentis* during the first 24 h is summarised in Table 5 and Fig. 11.

The majority of time-dependant DE defence-related genes between the ILL7537 resistant genotype and the ILL6002 susceptible genotype were found at 2 hpi, suggesting that the resistant genotype demonstrated an earlier and faster detection and signalling response to the *A. lentis* infection, thus being better prepared molecularly to deploy critical defence response proteins. In addition, overall higher expression levels of structural defence response genes were found in the resistant genotype regardless of the time post inoculation, indicating an innate ability to form stronger structural barriers against the fungus compared with the susceptible genotype.

The information provided by this study further extends the available knowledge of lentil resistance to *A. lentis* infection and may assist in future efforts to identify and develop additional resistant cultivars and management strategies, thereby reducing the losses caused by the pathogen.

## Additional files

Additional file 1 Trimmed Mean of M-values (TMM) of the estimated expression of selected defence-related genes in lentil–*A. lentis* transcriptome. Treatment – Mock (M) for H_2_O control, Treatment (T) for ALP2 *A. lentis* inoculation. HPI – Hours post inoculation. Norm_TMM_count – Trimmed Mean of M-values of the raw estimated counts, as calculated by edgeR. Count_SE – Standard error values per gene at each genotype/time point, calculated from TMM counts of subgroup replicates (*n* =3). (XLSX 28 kb)

Additional file 2 Detailed information and statistics of read and base trimming per sample read file. (XLSX 17 kb)

## Abbreviations

bp: Base pairs (measuring unit)
DE: Differential expression/differentially expressed
FC: Fold change
FDR: False discovery rate
GO: Gene Ontology
hpi: Hours post inoculation (measuring unit)
KEGG: Kyoto encyclopedia of genes and genomes
NCBI: National Center for Biotechnology Information
ORF: Open reading frame
PCR: Polymerase chain reaction
PR: Pathogenesis-related
QC: Quality check
RNA-Seq: RNA-sequencing
ROS: Reactive oxygen species
RQI: RNA quality indicator
RT-qPCR: Quantitative reverse transcription PCR
SRA: Sequence read archive
TMM: Trimmed mean of *m*-values
